# The Effects of Open-World and Fun, Accessible Games on Perceived Loneliness and Stoicism in Adults: Cross-Sectional Survey Study

**DOI:** 10.2196/89304

**Published:** 2026-06-17

**Authors:** Congcong Hou, Winze Tam, Andini Ayu Rahmadianty, Pradana Rajendra, Andreas Benedikt Eisingerich

**Affiliations:** 1 Faculty of Commerce, Kyushu Sangyo University, Fukuoka, Japan; 2 Imperial College London, London, United Kingdom; 3 Department of Analytics, Marketing and Operations, Imperial College London, South Kensington Campus, London, SW7 2AZ, United Kingdom, 44 207594 ext 9763, 44 2075949184

**Keywords:** video games, loneliness, stoicism, open-world games, fun games, Zelda, Yoshi

## Abstract

**Background:**

Loneliness has been linked to reduced mental and physical health. The “loneliness epidemic” is recognized as a public health crisis. However, questions remain about the potential of video games, which people play by themselves, to help reduce perceived loneliness.

**Objective:**

This study explored the extent to which open-world games (eg, *The Legend of Zelda: Breath of the Wild*) and fun, accessible games (eg, *Yoshi’s Crafted World*) can help reduce loneliness in adults. We examined how such gameplay can foster a stoic approach to life and how stoicism mediates the reduction of perceived loneliness.

**Methods:**

A cross-sectional survey was conducted using convenience sampling near a video game store. The sample comprised 2252 adults aged 21 years and older (women: n=966, 42.90%; men: n=1281, 56.90%; prefer not to disclose: n=5, 0.20%). Participants completed a questionnaire to measure perceived loneliness and stoicism, as well as their gameplay habits. Data were analyzed using ANOVA and moderated mediation with the PROCESS macro (bootstrapped samples=5000; 95% CI) to examine the effects of video gameplay on stoicism and loneliness, with the α level set at .05.

**Results:**

Zelda players indicated higher stoicism (mean 4.87, SD 0.11; 95% CI 4.66-5.08) than nonplayers (mean 3.23, SD 0.07; 95% CI 3.09-3.37; *F*_1,2252_=164.95; *P*<.001). Yoshi players also noted significantly higher stoicism (mean 4.49, SD 0.12; 95% CI 4.27-4.72) than nonplayers (mean 3.61, SD 0.05; 95% CI 3.50-3.71; *F*_1, 2252_=48.33; *P*<.001), with a significant interaction effect (*F*_1,2252_=7.89; *P*=.005) on stoicism. Furthermore, Zelda players indicated lower loneliness (mean 3.02, SD 0.11; 95% CI 2.81-3.22) than nonplayers (mean 4.28, SD 0.07; 95% CI 4.14-4.42; *F*_1, 2252_=98.32; *P*<.001). Yoshi players also noted significantly lower loneliness (mean 3.09, SD 0.12; 95% CI 2.86-3.32) than nonplayers (mean 4.21, SD 0.05; 95% CI 4.10-4.32; *F*_1, 2252_=76.32; *P*<.001). Moderated mediation analysis demonstrated that Zelda gameplay was positively associated with stoicism (β=1.28, 95% CI 1.07-1.50; *P*<.001), and stoicism was negatively associated with perceived loneliness (β=−0.49, 95% CI −0.52 to −0.45; *P*<.001).

**Conclusions:**

This study is innovative in identifying stoicism as a potential emotional pathway through which video games may reduce loneliness. Moving beyond views of gaming as passive escapism, our findings suggest that specific gameplay experiences may serve as active spaces for cultivating resilience. We introduce a “digital diet” framework, indicating that balancing open-world challenges (eg, Zelda) with low-stakes restoration (Yoshi) produces synergistic psychological support. Practically, thoughtfully curated gaming experiences may serve as scalable and cost-effective digital adjuncts for public mental health interventions addressing the loneliness epidemic.

## Introduction

### Overview

Addressing perceived loneliness is perhaps one of the most pressing psychosocial challenges of our time [1-7]. Its importance today is magnified by a unique confluence of technological [8,9], societal [10,11], and economic factors [12] that differentiate the modern experience of loneliness from that of any previous generation [13]. Critically, the “loneliness epidemic” is a recognized public health crisis [14-20]. Loneliness is no longer seen as just a fleeting feeling [21]; it is now understood to have severe consequences for both mental health [22,23] and physical health [24], on par with smoking and obesity [25,26].

Chronic loneliness is linked to a 26% increased risk of premature mortality [24,27]. It is associated with higher levels of cortisol (the stress hormone) [28], increased inflammation [29], weakened immune systems [30,31], high blood pressure [32], and heart disease [33]. Furthermore, loneliness is deeply detrimental to mental health [34,35], and is associated with depression, anxiety, and substance abuse [36-40]. It can create a negative feedback loop where loneliness leads to increased social anxiety [21] and negative perceptions of social interactions [24], which, in turn, lead to further isolation [38]. Addressing loneliness is, therefore, a critical public health priority [21,37]; mitigating it can reduce the burden on health care systems and improve overall population well-being.

Despite society existing in the most technologically connected age [21], modern social platforms often promote performative interactions rather than authentic vulnerability, fostering feelings of inadequacy or “FOMO” (Fear of Missing Out) [41] or “FOBO” (Fear of a Better Option Elsewhere). Concurrently, participation in traditional localized communities has declined [42]. Modern, individualized lifestyles [43] and high-stress “hustle” cultures can crowd out the energy required to nurture relationships [42], increasingly leading to a sense of existential isolation [44].

In prior research, the role of video games in relation to loneliness has yielded mixed findings. On one hand, loneliness has been associated with problematic gaming outcomes, such as internet gaming disorder, in some populations [18]. On the other hand, socially oriented gaming interventions have been explored as a possible way to reduce loneliness, particularly among older adults [6]. However, what remains uncertain is the potential for mainstream single-player game genres, specifically open-world and fun, accessible games, to mitigate loneliness internally. While existing work has begun to examine the social potential of multiplayer or socially oriented gaming, the current literature lacks a clear understanding of the internal cognitive and emotional pathways through which specific single-player game environments might alleviate perceived loneliness without relying on digital socializing.

### Background on Stoicism and Loneliness

In the context of performative connection and external pressure, stoicism can offer a counterintuitive but powerful solution. Stoicism does not simply advocate to “meet more people.” It addresses the root perception of lack [45]. That is, stoicism shifts the source of validation from external to internal [45-47]. If an individual’s sense of worth comes from their own character and virtues, they are less vulnerable to the approval and curated feeds of others [45-48]. Critically, stoicism helps reframe solitude, transforming loneliness into purposeful solitude—a time for reflection, self-improvement, and cultivating inner peace [45]. When one is comfortable with oneself, one can attract healthier connections and engage with the world from a place of strength, not lack.

While theoretical frameworks relevant to endurance, self-regulation, and responses to adversity can be found in stoic writings [45-48], direct quantitative evidence examining the association between stoicism and lower levels of loneliness remains limited. However, empirical support exists for some of the psychological mechanisms often linked to stoic practice. For example, cognitive behavioral therapy has frequently been connected to stoic principles, particularly the role of cognition in shaping emotional experience [49,50], and a cognitive behavioral therapy–based intervention has been shown to reduce loneliness in a randomized controlled trial [16]. Furthermore, recent psychometric research conceptualizes philosophical stoicism as a specific, measurable set of attitudes and behaviors rather than a strictly fixed personality trait [51]. While intervention studies explicitly targeting stoicism remain rare, evidence from clinical settings suggests that cognitive processes closely aligned with stoic practice, such as appraisal and reinterpretation, may be malleable [16,49]. Thus, a secondary aim of this study is to address this empirical gap by providing quantitative evidence on whether self-reported stoic attitudes are negatively associated with perceived loneliness.

Stoicism teaches individuals to direct their energy only toward things within their control (their judgments, values, and actions) and to accept what they cannot control (external events, other people’s actions). The practice of accepting and embracing everything that happens (*amor fati* or love of fate) is viewed not as a setback but as a necessary part of life’s journey [45,47,48]. According to stoicism, true happiness (*eudaimonia*) comes from living a life of virtue (wisdom, courage, justice, and temperance), not from seeking external pleasure or avoiding pain [45]; it is also seen as a path toward mental freedom [52,53]. In addition to this, contemplating potential setbacks (*premeditatio malorum* or the premeditation of evils) can help reduce their emotional impact and prepare individuals for adversity [45,46]. Stoicism aims for a state of *ataraxia* (freedom from distress) [45,46], offering a recalibration of one’s emotional baseline to recognize that peace and joy are possible internal states, independent of external circumstances. Finally, a core stoic exercise is *memento mori* (“remember you must die”), not to be morbid, but to appreciate the preciousness of life and to prioritize what truly matters [45-47]. That is, to appreciate the beauty of something precisely because it is fleeting.

### Background on Video Gameplay, a Stoic Mindset, and Loneliness

#### Video Gameplay and Loneliness

Prior empirical research demonstrates that video games can positively impact mental well-being and provide experiences of relaxation and cognitive escapism [54,55]. Furthermore, broader reviews indicate that game-based digital interventions can reduce mental health symptoms in clinical contexts [56,57]. Building on such evidence of gaming’s capacity to enhance mastery and overall well-being, we posit that the structured environments of open-world and fun, accessible games could potentially serve as a novel context for cultivating attitudes aligned with stoic philosophy.

By design, open-world games often place players in vast, indifferent environments where survival requires adaptability and resilience. *The Legend of Zelda: Breath of the Wild* serves as a prime, concrete example of this dynamic, as it forces players to embrace a harsh, often unforgiving environment. This is a direct practice of *amor fati*.

In addition, gameplay emphasizes self-reliance and internal resources (virtue as the sole good). Link begins the game with nothing. He is amnesiac, weak, and stripped of all past glory. Progress in the game is not handed to anyone. Players must cook their own meals, forage for ingredients, scavenge for weaker weapons, and slowly learn to conquer challenges through their own wits, courage, and perseverance. The game constantly reinforces the idea that one has the resources within oneself to overcome obstacles. This builds self-sufficiency, a core stoic value. Players learn to rely on their judgment and abilities rather than waiting for rescue or a lucky break.

Moreover, the game’s core loop is exploration and observation, not just action. Stoicism begins with the “discipline of perception,” which involves judging events clearly and without unnecessary emotional baggage.

Furthermore, accessible, low-stakes games, such as Yoshi, can help strengthen the stoic focus on the present moment through mindfulness and simplicity. The mindfulness is baked into the esthetic and gameplay. *Yoshi’s Crafted World* is hand-crafted from cardboard and yarn, encouraging you to appreciate the beauty in simple, everyday objects. The pace is slower, and the goal is often to stop, look around, and find a hidden flower or a shy Poochy pup. This is a practice in appreciating the present moment for its own sake, not just for survival. For someone feeling lonely, this directly counteracts rumination about the past or anxiety about the future, anchoring them in a gentle, pleasant now.

Moreover, games such as *The Legend of Zelda: Breath of the Wild* and *Yoshi’s Crafted World* reinforce the idea that purpose is often found in caring for others and contributing to a collective good. They practice the stoic concept of *sympatheia*, the interconnectedness of all people [45]. By engaging in a game that is literally about helping and supporting others (even in a simple, cartoonish way), players can strengthen the neural pathways for empathy and the understanding that we are part of a whole. This directly combats the isolated, ego-centric perception that fuels loneliness.

Loneliness is often less about being physically alone and more about a state of mind characterized by a feeling of lack or neediness, a sense of disconnection from the world and others, as well as anxiety about one’s place and ability to cope. The stoic mindset cultivated by *The Legend of Zelda: Breath of the Wild* can help directly address these feelings. That is, the game helps reframe solitude from a state of *loneliness* to one of peaceful aloneness, self-reliance, and agency. The quiet, expansive landscapes are not empty; they are full of possibility that one gets to engage with on one’s own terms. This teaches players to enjoy their own company and to see alone time not as a deficiency, but as an opportunity for exploration and self-discovery. One becomes a complete unit unto oneself, less dependent on others for validation or entertainment.

Stoicism teaches that people are part of a larger, rational cosmos. *The Legend of Zelda: Breath of the Wild* mimics this by fostering a connection to something larger and by making players a small part of a vast, open world. One’s journey is one’s own, but it is woven into the fate of Hyrule. This sense of being part of a larger whole, even when alone, can be profoundly comforting and diminish the feeling of being a lonely, isolated individual.

#### The Perfect Stoic Diet

Playing *The Legend of Zelda: Breath of the Wild* builds the core, resilient stoic muscles: courage, endurance, acceptance, and self-reliance. Playing a Yoshi game rehabilitates those muscles with stretches of joy, nurtures them with empathy, and keeps them flexible with mindfulness and play. Together, they form a holistic practice. Open-world games such as *The Legend of Zelda: Breath of the Wild* teach players to be strong enough to be alone without being lonely. Fun and accessible games such as Yoshi, on the other hand, can remind one that the world is also a place of softness, connection, and simple joys worth engaging with. Together, these games remind us that light-heartedness and profundity are not opposites but essential partners on the path to a good life. By becoming more stoic, one becomes more resilient, self-contained, and at peace with one’s own presence. This inner fortitude is the ultimate cure for loneliness, as it means one is never truly without a stable and capable companion: oneself.

Thus, the aim of this study is to investigate the potential of open-world and fun, accessible video games for reducing loneliness and enhancing stoicism in adults. To achieve this, we address four distinct but interconnected research questions: First, to what extent, if at all, can video gameplay help reduce the risk of loneliness? Second, how does a stoic approach to life, afforded by these games, serve as an emotional pathway to alleviate that isolation? Third, what role do popular open-world video games play in facilitating this resilient stoic mindset? Finally, how does the addition of fun, accessible games (eg, *Yoshi’s Crafted World*) interact with open-world gameplay (eg, *The Legend of Zelda: Breath of the Wild*) to further enhance stoicism and ultimately mitigate people’s loneliness?

## Methods

### Research Design

As part of this study, we conducted a cross-sectional survey using a convenience sampling approach. We followed the STROBE (Strengthening the Reporting of Observational Studies in Epidemiology) guidelines (Checklist 1) to report observational, cross-sectional studies [58] and collected the data in November 2025 and December 2025.

### Inclusion and Exclusion

**Figure 1.**
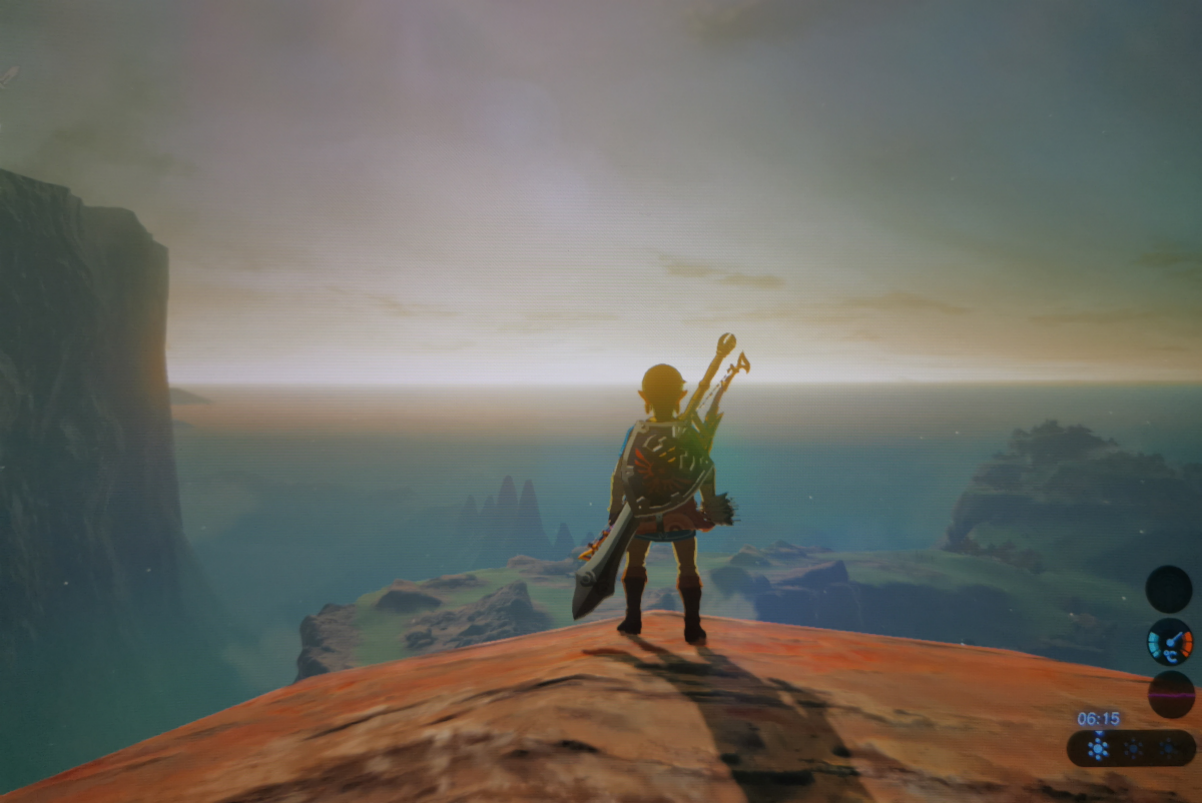
Link, ready to keep exploring as the sun rises in *The Legend of Zelda: Breath of the Wild* (reproduced with permission from Nintendo).

**Figure 2.**
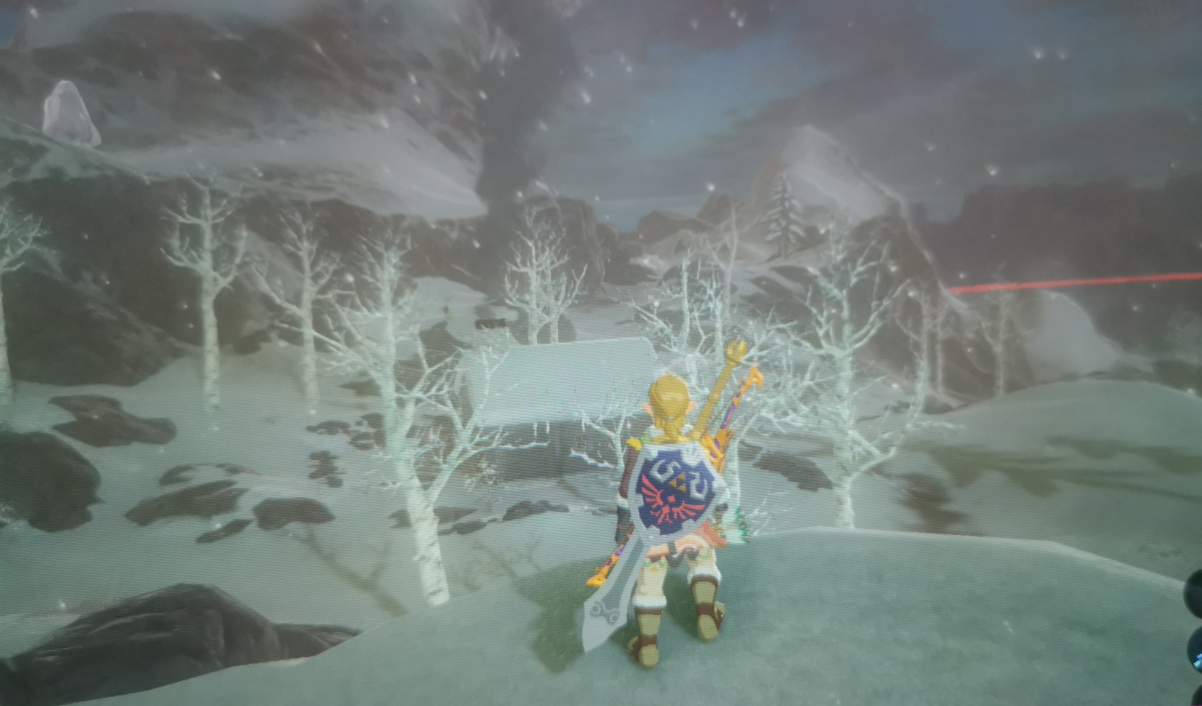
Hebra Mountains and their unforgiving freezing climate in *The Legend of Zelda: Breath of the Wild* (reproduced with permission from Nintendo).

**Figure 3.**
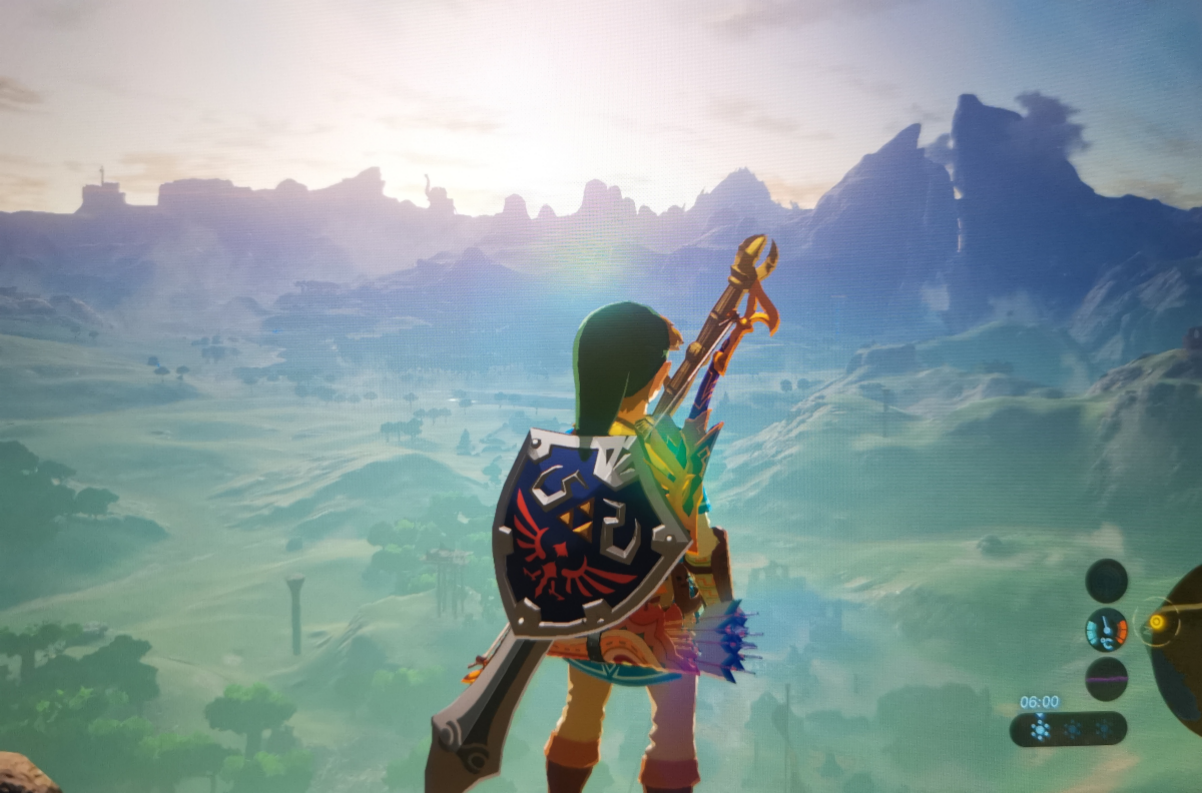
Link overlooking parts of the vast, open world of Hyrule in *The Legend of Zelda: Breath of the Wild* (reproduced with permission from Nintendo).

**Figure 4.**
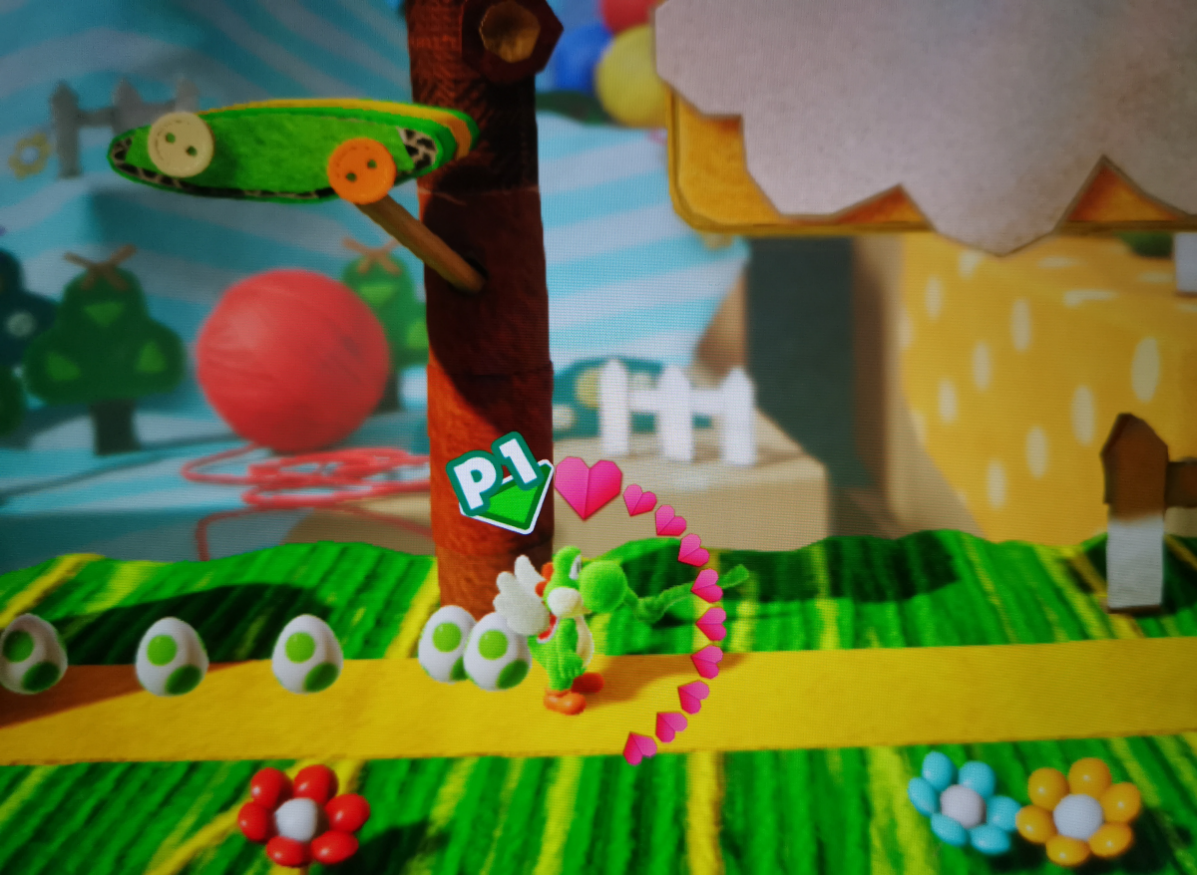
Cheerful landscape in *Yoshi’s Crafted World* (reproduced with permission from Nintendo).

Participants were invited to take part in the study if they were aged 21 years or older. To allow for robust statistical comparisons, prior or current video game experience was not required. Specifically, we compared nonplayer participants with participants who currently or recently played open-world games (eg, *The Legend of Zelda: Breath of the Wild* or *The Legend of Zelda: Tears of the Kingdom*; see Figures 1-3 for visual context of the vast, open-world environments that require a stoic approach) and/or fun, accessible games (eg, *Yoshi’s Crafted World* or *Yoshi’s Wooly World*; see Figures4  5 for the gentle, nurturing environments).

**Figure 5.**
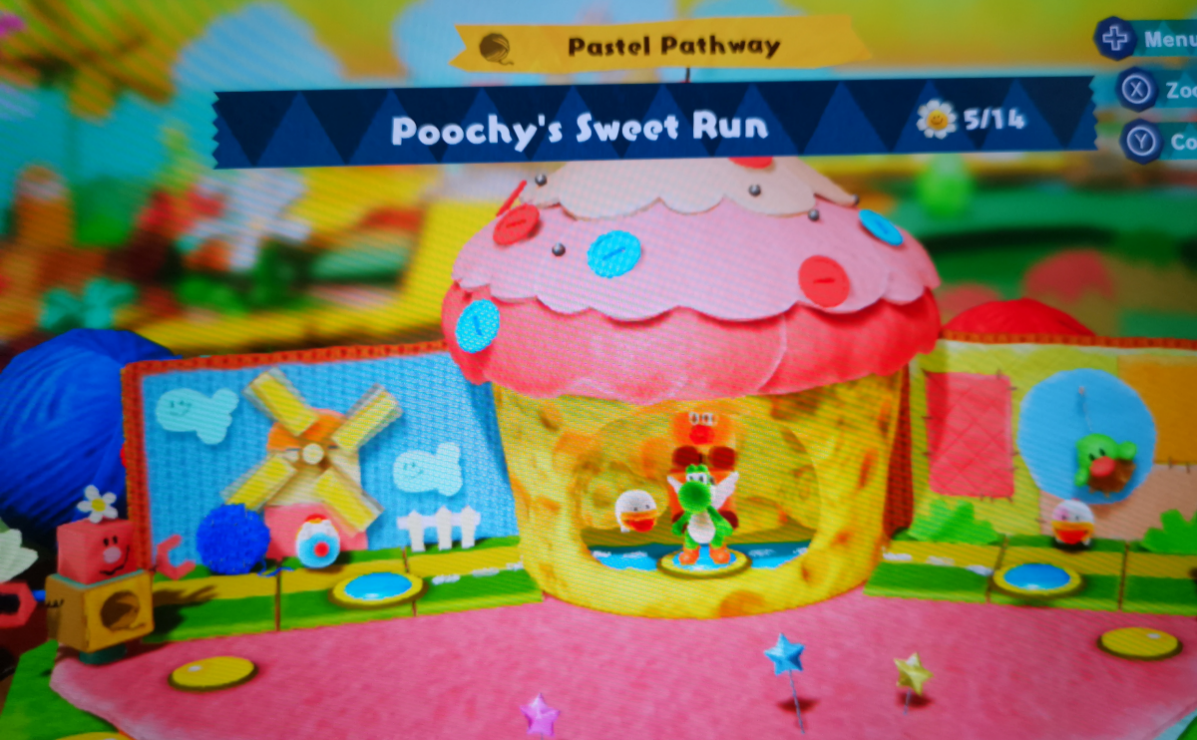
Heartwarming environment in *Yoshi’s Crafted World* (reproduced with permission from Nintendo).

### Sampling Procedures

A total of 3 trained research assistants, who were blind to the study’s research questions, approached potential study participants near a video game store in a busy shopping mall. This was done to capture some variance in terms of whether people were playing or had recently played an open-world game such as *The Legend of Zelda: Breath of the Wild* or *The Legend of Zelda: Tears of the Kingdom* and/or fun, accessible games such as *Yoshi’s Crafted World* or *Yoshi’s Wooly World*. The research assistants informed potential participants that the study, which focused on daily activities, would contribute to current academic research at a university and that participation would not take longer than 7 minutes in total. The research assistants also checked participants’ IDs for age in cases where potential participants appeared younger.

### Sample Size, Power, and Precision

Over the course of 5 weeks, the research assistants managed to recruit a total of 2300 participants and invited them to complete a brief survey on a tablet. Because 48 participants stopped midway through answering the measurement items and, hence, did not complete the entire questionnaire, their responses were discarded from the study, and the total effective response rate is 2252 (97.90% completion rate).

### Participant Characteristics

The final sample consisted of 2252 participants (women: n=966, 42.90%; men: n=1281, 56.90%; prefer not to disclose sex: n=5, 0.20%). Because participants with incomplete responses were excluded during the initial screening phase, there were no missing data in the final analyzed dataset.

### Measures and Covariates

Participants answered 3 questions about perceived loneliness that were adapted from previously published and validated scales [59-61]: “How often do you feel a sense of emptiness around you?”, “How often do you feel that there is no one you can turn to?”, and “How often do you feel that you lack companionship?” (1=“never” to 7=“always”). Furthermore, participants answered 3 questions about their stoic attitudes and behaviors using items that were adapted from previously published and validated scales designed to measure the core practices of modern stoicism [49-51], “I reflect on the temporary nature of things to help me appreciate them more,” “I consciously consider whether things are within my control before deciding how to act,” and “When facing a difficulty, I focus on my own virtuous response rather than complaining about the situation itself” (1=“very inaccurate” to 7=“very accurate”). Participants then indicated whether they are currently playing or have recently played a Yoshi game such as *Yoshi’s Crafted World* or *Yoshi’s Wooly World* (0=“no” and 1=“yes”), and an open-world game from the Legend of Zelda series, such as *The Legend of Zelda: Breath of the Wild* or *The Legend of Zelda: Tears of the Kingdom* (0=“no” and 1=“yes”). As a control, participants indicated their gender (0=“female” and 1=“male”). To ensure the quality of measurement, all scale items were reviewed by the research team for clarity and consistency.

### Statistical Analysis

We followed the recommended procedures for conducting confirmatory factor analyses and examined the factor structure and reliability scores for all the measurement items in our survey study [62,63]. This was done to assess whether the items in the study significantly loaded on their intended factors and had weak cross-loadings. Furthermore, we examined the average variance extracted and composite reliability scores for the measurement scales in our study. Moreover, we tested whether the average variance extracted for each construct was indeed greater than the squared correlations between the construct and other variables in our survey [64]. The hypothesized model was tested using confirmatory factor analysis in IBM SPSS AMOS 29.0 with maximum likelihood estimation.

Next, we conducted an ANOVA to examine the variances across the different groups in our study (ie, open-world game: no vs yes; fun, accessible game: no vs yes). In addition, we conducted a moderated mediation analysis using the PROCESS macro [65] (model 8; bootstrapped samples=5000; 95% CI) to examine the effects of popular open-world gameplay such as *The Legend of Zelda: Breath of the Wild* (independent variable) as well as gameplay of fun, accessible games, such as *Yoshi’s Crafted World* (moderator) on stoicism (mediator) and loneliness (dependent variable). To ensure that PROCESS did not treat sex as a continuous variable, sex was coded as a dummy variable (0=“female” and 1=“male”). Due to the insufficient sample size (n=5), the “prefer not to disclose” category was excluded from the moderated mediation analysis.

### Ethical Considerations

Ethical approval for this study was obtained from Kyushu Sangyo University’s ethics committee (#2025‐0019). The procedure ensured compliance with the university’s research ethics guidelines and the principles of the Declaration of Helsinki. All data collected were anonymized and kept confidential within the research team. Study participants provided informed consent and were told that they had the right to withdraw from the study at any time without providing a specific reason for withdrawal. Furthermore, we ensured that no identification of individual participants or users in any images included in the paper (eg, in-game screenshots and participant flowchart) or supplementary material was possible. After completing the brief survey, each study participant was thanked for their time and debriefed about the study. Study participants did not receive a financial reward.

## Results

### Participant Characteristics

**Figure 6.**
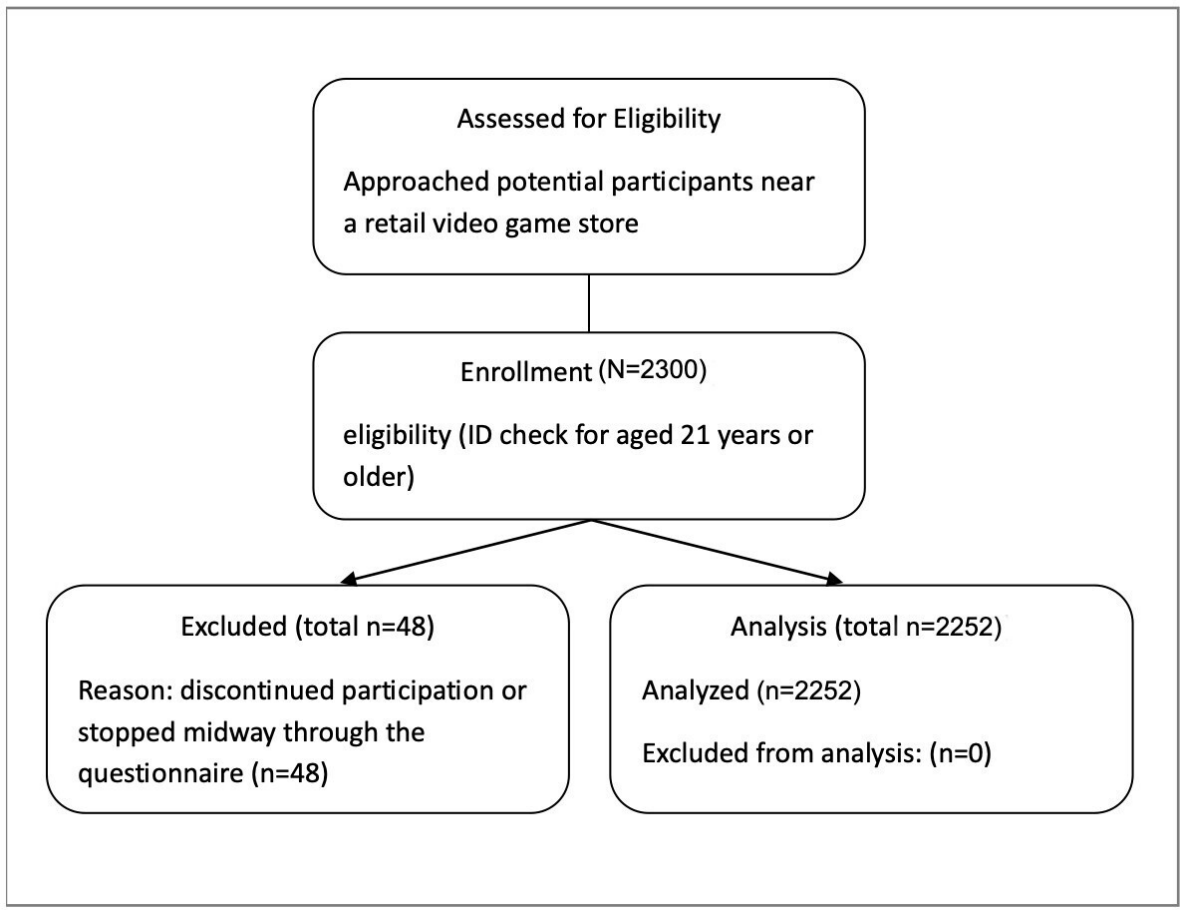
Flow of participants through the screening, enrollment, and analysis stages in the cross-sectional survey study.

The demographic and baseline gameplay characteristics of the sample are detailed in Table 1 and Figure 6.

**Table 1.** Participant characteristics (N=2252).

Characteristic	Participants, n (%)
Sex	
Women	966 (42.90)
Men	1281 (56.90)
Prefer not to disclose	5 (0.20)
Gaming experiences	
Open-world gameplay (eg, *The Legend of Zelda: Breath of the Wild*)	
Yes	416 (18.50)
No	1836 (81.50)
Fun, accessible gameplay (eg, *Yoshi’s Crafted World*)	
Yes	263 (11.70)
No	1989 (88.30)

### Statistics and Data Analysis: Measurement Model

Table 2 lists the detailed measurement items used in this study as well as their factor loadings and reliabilities. Confirmatory factor analysis results demonstrated that all the measurement items in this study significantly loaded on their intended factors (>0.88; *P*<.001) with weak cross-loadings (<0.28).

**Table 2.** Cross-sectional survey measurement items’ factor loadings and reliability scores.

Constructs and measurement items	Factor loadings
Loneliness (*α*=.93; CR^a^=.92; AVE^b^=.81)	
How often do you feel a sense of emptiness around you?	0.89
How often do you feel that there is no one you can turn to?	0.91
How often do you feel that you lack companionship?	0.90
Stoicism (*α*=.94; CR=.93; AVE=.83)	
I reflect on the temporary nature of things to help me appreciate them more	0.92
I consciously consider whether things are within my control before deciding how to act	0.88
When facing a difficulty, I focus on my own virtuous response rather than complaining about the situation itself	0.93

a CR: composite reliability.

b AVE: average variance extracted.

### Primary Analyses: ANOVA

A set of 2 (Zelda_open-world gameplay_: no vs yes)×2 (Yoshi_fun, accessible gameplay_: no vs yes) ANOVA on stoicism showed a significant main effect of open-world gameplay such that participants who played an open-world game like *The Legend of Zelda: Breath of the Wild* indicated higher levels of stoicism (mean 4.87, 95% CI 4.66-5.08) than participants who did not (mean 3.23, 95% CI 3.09-3.37; *F*_1, 2252_=164.95; *P*<.001). Moreover, the results demonstrated a significant main effect of fun, accessible gameplay, such that participants who played a Yoshi game noted significantly higher levels of stoicism (mean 4.49, 95% CI 4.27-4.72) than study participants who did not (mean 3.61, 95% CI 3.50-3.71; *F*_1, 2252_=48.33; *P*<.001). In addition to this, we observed a significant interaction effect of open-world× fun, accessible gameplay (*F*_1, 2252_=7.89; *P*=.005). Gender had no effect on stoicism (*F*_1, 2252_=0.01; *P*=.91; Multimedia Appendix 1).

Furthermore, we conducted a set of 2 (Zelda_open-world gameplay_: no vs yes)×2 (Yoshi_fun, accessible gameplay_: no vs yes) ANOVA on loneliness. The results showed a significant main effect of open-world gameplay, such that participants who played an open-world game like *The Legend of Zelda: Breath of the Wild* indicated lower levels of loneliness (mean 3.02, 95% CI 2.81-3.22) than participants who did not (mean 4.28, 95% CI 4.14-4.42; *F*_1, 2252_=98.32; *P*<.001). In addition, the results showed a significant main effect of fun, accessible gameplay such that participants who played a Yoshi game noted significantly lower levels of loneliness (mean 3.09, 95% CI 2.86-3.32) than study participants who did not (mean 4.21, 95% CI 4.10-4.32; *F*_1, 2252_=76.32; *P*<.001). The results also showed a significant interaction effect of open-world× fun, accessible gameplay (*F*_1, 2252_=4.78; *P*=.03), while gender had no effect on loneliness (*F*_1, 2252_=0.34; *P*=.56; Multimedia Appendix 2).

### Primary Analyses: Moderated Mediation

We conducted moderated mediation analyses using PROCESS macro [65] (model 8; bootstrapped samples=5000; 95% CI; sample size=2252) with Zelda_open-world gameplay_ as the independent variable, stoicism as the mediator, Yoshi_fun, accessible gameplay_ as the moderator, and loneliness as the dependent variable with gender as a potential confound. The results showed that Zelda_open-world gameplay_ (independent variable) had a significant and positive effect on stoicism (β=1.28, SE=0.11, *t*=11.83; *P*<.001; 95% CI 1.07-1.50; Table 3). Furthermore, Yoshi_fun,accessible gameplay_ had a positive influence on stoicism (β=0.53, SE=0.14, t=3.70; *P*<.001; 95% CI 0.25-0.81) and the results showed a significant interaction effect of Zelda and Yoshi gameplay on stoicism (β=0.72, SE=0.26, *t*=2.81; *P*=.005; 95% CI 0.22-1.22). Gender had no influence on stoicism (β=0.01, SE=0.08, *t*=0.11; *P*=.91; 95% CI –0.14 to 0.16; Table 3).

**Table 3.** Cross-sectional survey moderated mediation regression results: effects of open-world and fun, accessible gameplay on stoicism^a^.

Predictor	B	SE	*t* test	*P* value	95% CI
DV: Stoicism					
Constant	2.95	0.13	23.39	<.001	2.70 to 3.20
Zelda_open-world gameplay_	1.28	0.11	11.83	<.001	1.07 to 1.50
Yoshi_fun, accessible gameplay_	0.53	0.14	3.70	<.001	0.25 to 0.81
Interaction Zelda × Yoshi gameplay	0.72	0.26	2.81	.005	0.22 to 1.22
Gender	0.01	0.08	0.11	.91	–0.14 to 0.16

a *R*=.33; *R*²=.11; mean square error=3.19; *F*_4, 2247_=72.37*; P*<.001.

The conditional effects of the focal predictor (open-world gameplay such as *The Legend of Zelda: Breath of the Wild*) on stoicism at values of the moderator (fun, accessible gameplay such as Yoshi: no vs yes) are shown in Table 4.

**Table 4.** Cross-sectional survey conditional effects: effects of the focal predictor (Zelda_open-world gameplay_) on stoicism at values of the moderator (Yoshi_fun, accessible gameplay_).

	Effect	SE	*t* test	*P* value	95% CI
Zelda -> stoicism					
Yoshi gameplay: no	1.28	0.11	11.83	<.001	1.07-1.50
Yoshi gameplay: yes	2.00	0.23	8.64	<.001	1.55-2.46

The moderated mediation analyses further showed that stoicism was significantly negatively associated with participants’ perceived loneliness (β=−0.49; SE=0.02; *t*=−26.46; *P*<.001; 95% CI −0.52 to −0.45; Table 5). In addition, the direct effects of Zelda and Yoshi gameplay on loneliness remained significant such that Zelda (β=-0.36; SE=0.10; *t*=−3.72; *P*<.001; 95% CI −0.56 to −0.17) and Yoshi (β=−0.58; SE=0.13; *t*=−4.62; *P*<.001; 95% CI −0.83 to −0.33) gameplay were significantly negatively associated with loneliness (Table 5). The interaction of Zelda×Yoshi gameplay (β=−0.21; SE=0.22; *t*=−0.93; *P*=.35; 95% CI −0.65 to 0.23) on loneliness was not significant and the results showed no effect of gender on loneliness (β=−0.04; SE=0.07; *t*=−0.60; *P*=.55; 95% CI −0.17 to 0.09; Table 5).

**Table 5.** Cross-sectional survey moderated mediation regression results: effects of open-world and fun, accessible gameplay on loneliness^a^.

Predictor	B	SE	T	*P* value	95% CI
DV: Loneliness					
Constant	6.21	0.12	50.51	<.001	5.97 to 6.45
Zelda_open-world gameplay_	−0.36	0.10	−3.72	<.001	−0.56 to –0.17
Stoicism	−0.49	0.02	−26.46	<.001	−0.52 to –0.45
Yoshi_fun, accessible gameplay_	−0.58	0.13	−4.62	<.001	−0.83 to –0.33
Interaction Zelda × Yoshi gameplay	−0.21	0.22	−0.93	.35	−0.65 to 0.23
Gender	−0.04	0.07	−0.60	.55	−0.17 to 0.09

a *R*=.56; *R*²=.31; mean square error=2.44; *F*_5, 2246_=201.60; *P*<.001.

Table 6 shows the conditional direct effects of the focal predictor (open-world gameplay, such as *The Legend of Zelda: Breath of the Wild*) on loneliness at values of the moderator (fun, accessible gameplay, such as Yoshi: no vs yes). The conditional indirect effects of open-world gameplay, such as *The Legend of Zelda: Breath of the Wild*, on loneliness at values of the moderator (fun, accessible gameplay such as Yoshi: no vs yes) are shown in Table 7.

**Table 6.** Cross-sectional survey conditional direct effects: effects of the focal predictor (Zelda_open-world gameplay_) on loneliness at values of the moderator (Yoshi_fun, accessible gameplay_).

	Effect	SE	T	*P* value	95% CI
Zelda -> loneliness					
Yoshi gameplay: no	−0.36	0.10	−3.72	<.001	−0.56 to −0.17
Yoshi gameplay: yes	−0.57	0.21	−2.78	.006	−0.98 to −0.17

**Table 7.** Cross-sectional survey conditional indirect effects: effects of Zelda_open-world gameplay_ on loneliness at values of the moderator (Yoshi_fun, accessible gameplay_).

	Effect	BootSE	95% CI (BootLLCI-BootULCI)
Zelda -> stoicism -> loneliness			
Yoshi gameplay: no	−0.63	0.06	−0.74 to −0.51
Yoshi gameplay: yes	−0.98	0.10	−1.18 to −0.79

## Discussion

### Principal Findings

In alignment with our stated objectives, this study investigated the potential of open-world and accessible video games to reduce perceived loneliness and enhance stoicism in adults. Addressing our four core research questions, the findings demonstrated that, first, engagement with these focal games is significantly and negatively associated with perceived loneliness. Second, our theoretical model revealed that stoicism serves as a significant emotional pathway, inversely linking gameplay to feelings of isolation. Third, open-world gameplay (eg, The Legend of Zelda) was strongly associated with a more resilient, stoic mindset. Finally, we observed a significant interaction effect, indicating that the addition of accessible games (eg, Yoshi) positively synergizes with open-world gameplay to further enhance stoicism.

The observed synergy between open-world and accessible gameplay, termed the “Perfect Stoic Diet,” suggests potential as a scalable public health intervention. Traditional mental health services often contend with well-documented barriers, including broad gaps in care availability [66], cost and access challenges [67], and stigma related to mental illness [68]; in contrast, these gaming experiences are widely accessible [54,56]. While the upfront costs of commercial video games and required hardware present a tangible barrier for some vulnerable populations, broader research supports the idea that game-based digital interventions may offer scalable and relatively cost-effective adjuncts to conventional therapy, particularly for individuals from at-risk populations who might otherwise remain underserved [56,57]. Leveraging the immersive nature of these games to foster a stoic mindset provides a novel, decentralized pathway for addressing the loneliness epidemic [21,37].

### Comparison With Prior Work

Prior research examined video games and digital environments through the lens of cognitive escapism, relaxation, and mental well-being [54,55], including discussions on bridging gaming and mental health science [69]. Broader digital well-being research further shows that online environments can shape anxiety, stress, and psychological experiences in complex ways, depending on platform features, user goals, privacy concerns, and social dynamics [70-75]. Prior studies also investigated how various digital offerings can help users by offering them hope and making their lives easier [76-78]. However, the specific role of single-player video games as active spaces for cultivating emotional resilience remains underdeveloped. Our findings extend this literature by suggesting that open-world environments, such as *The Legend of Zelda: Breath of the Wild*, may afford more than passive escapism: in our data, they were associated with a stoic mindset that helped players reframe solitude.

Furthermore, while some loneliness-focused gaming interventions have explored social or multiplayer experiences, particularly among older adults [6], this study contributes a novel perspective by suggesting that single-player, mainstream games may mitigate loneliness through internal cognitive processes rather than digital socializing. By linking gameplay to stoic principles [45-48], this study offers quantitative evidence that specific single-player gameplay experiences may serve as an internal emotional pathway for alleviating perceived loneliness.

Finally, while public health research emphasizes the serious physical and mental health consequences of loneliness [14,21,25], game-based digital intervention research has only recently begun to examine how different genres may produce distinct psychological affordances [56,57,79]. Our research introduces the concept of a “digital diet,” suggesting that the psychological benefits of gaming are not monolithic. Specifically, our findings indicate that balancing open-world challenge and autonomy with the low-stakes, calming qualities of accessible games, such as *Yoshi’s Crafted World*, produces a synergistic pattern of psychological support. This extends previous frameworks of digital well-being by suggesting that a thoughtfully curated combination of gameplay experiences may offer a more robust buffer against loneliness than singular game genres alone.

### Limitations

As part of this study, we explored the role of open-world and fun, accessible games in facilitating stoicism and reducing loneliness. Future work studying the potential influence of other types of games on adults’ loneliness is richly deserving. Moreover, we invite additional work to conduct randomized controlled studies across different participant pools—explicitly accounting for unmeasured confounding variables such as socioeconomic status (eg, income level, educational attainment, and working conditions), marital status, living arrangements, and baseline social contact—to establish causality and help strengthen confidence in the generalizability of the current results. In addition, we did not account for participants’ varied lifestyles or capture their social media usage as part of this study. We invite future work to explore how social media usage and individuals’ lifestyles (eg, taking regular walks in parks, etc) may influence loneliness in tandem with video gameplay. Additionally, we captured self-reported levels of stoicism. A promising avenue for future research is to investigate objective measures of stoicism and assess its role in the relationship between gaming and loneliness.

Furthermore, it is important to acknowledge potential confounding variables related to our sample composition. Because we did not collect data on the general gaming habits of participants who answered “no” to playing the focal games (ie, The Legend of Zelda or Yoshi), it remains unclear whether this control group consisted of strictly nongamers or players of other video game genres. Similarly, while our sample consisted of adults aged 21 years and older, the differing target demographics and inherent appeal of open-world versus fun and accessible games may introduce subtle confounding factors. Capturing participants’ complete digital diets is a necessary step for future research to isolate these variables more effectively.

Finally, it is important to note the limitations regarding our analytical approach. Because we conducted a cross-sectional survey and all variables were measured at the same time, the temporality of the variables cannot be firmly established. Consequently, the mediation analyses may be considered exploratory. Furthermore, the assumptions regarding the absence of confounding among these variables must be interpreted with caution. Since we were not able to collect data on various demographic information of participants and other potential confounding variables, we invite future research to investigate some of these additional variables that may lead to important insights. We strongly encourage future studies to conduct longitudinal observations to better establish the temporality and relational dynamics of these variables.

### Future Research

This study has a number of limitations, which also offer promising paths for additional work. Previous research has shown that users may at times feel uneasy about different digital offerings [80-82], while at the same time being able to incorporate digital solutions as part of their concept of self if and when these offerings are seen as enabling their lives and making their lives easier [83]. Future studies that further explore the role of video games in making users’ lives easier and helping them through the day by affording emotional reactivity are very promising. In addition to this, we invite work to explore the potential for video games in enhancing individuals’ mental health and broader well-being by offering benefits that resemble relaxation, restoration, or meditative forms of psychological recovery [54,55]. Moreover, an increasing number of people may feel overwhelmed by the burden of modern life (24/7 news cycles, an “always on” social media culture, etc) and may lose the ability for childlike wonder and joy [56,57,66-68,79,84], while becoming increasingly detached from nature [85,86]. Yet, people also strive for meaning in their lives [87]. The current findings show how a game can be more than entertainment, how ancient wisdom can be applied to modern problems [45,50], and how interactive experiences may help cultivate a life with less loneliness and more meaning [44,87]. How fun and accessible video gameplay may interact with gameplay of open-world games to help players anchor more strongly in day-to-day life and help them manage daily worries and discomfort is worthy of further investigation [54,79]. Thus, we invite future research to help bridge gaming and mental as well as physical health science to help individuals create a personal, life-affirming way to live such that if a demon told them that they would have to live this exact same life, with all its pain and joy, over and over again for eternity, they would react not with despair but rather with triumphant joy [88].

### Conclusions

The findings of this study highlight the potential for video games to help combat loneliness. First, this study is innovative in identifying stoicism as an active emotional pathway through which video games may reduce loneliness. Second, unlike existing studies that view gaming primarily as passive escapism, our findings suggest that video games can serve as active spaces for cultivating mental resilience. Third, it introduces a novel ‘digital diet’ framework to the field, showing that balancing open-world challenges (eg, The Legend of Zelda) with low-stakes restoration (eg, Yoshi) produces a synergistic pattern of psychological support. Finally, regarding broader real-world implications, these findings position thoughtfully curated video games as scalable and cost-effective digital adjuncts for public mental health interventions addressing the loneliness epidemic [21,37].

## Supplementary material

10.2196/89304 Multimedia Appendix 1 Effects of playing open-world and accessible or fun games on stoicism (mean scores).

10.2196/89304 Multimedia Appendix 2 Effects of playing open-world and accessible or fun games on perceived loneliness (mean scores).

10.2196/89304 Checklist 1 STROBE checklist.

## Abbreviations

STROBE: Strengthening the Reporting of Observational Studies in Epidemiology
