# Supplementary figures and images for: The Effects of Open-World and Fun, Accessible Games on Perceived Loneliness and Stoicism in Adults: Cross-Sectional Survey Study
Source: JMIR Serious Games. 2026 Jun 17;14:e89304. doi: 10.2196/89304 (PMC13274968; doi:10.2196/89304)

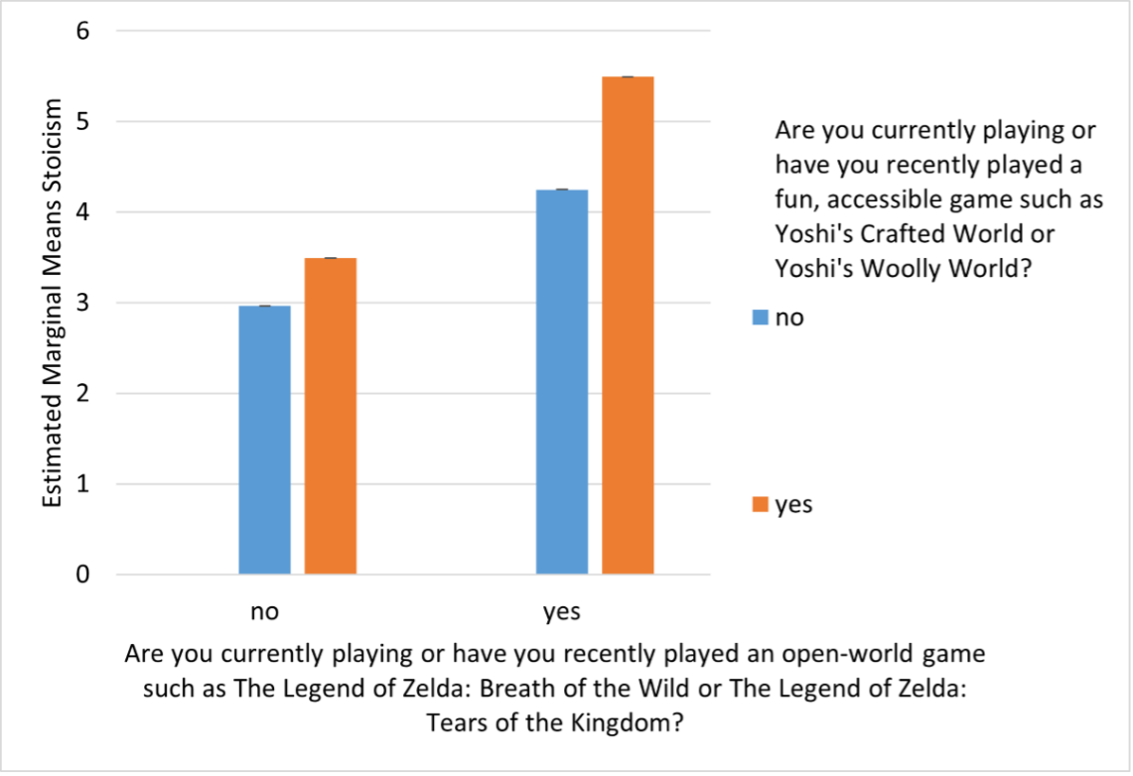

Supplement: Multimedia Appendix 1 [file games-v14-e89304-s001.png]

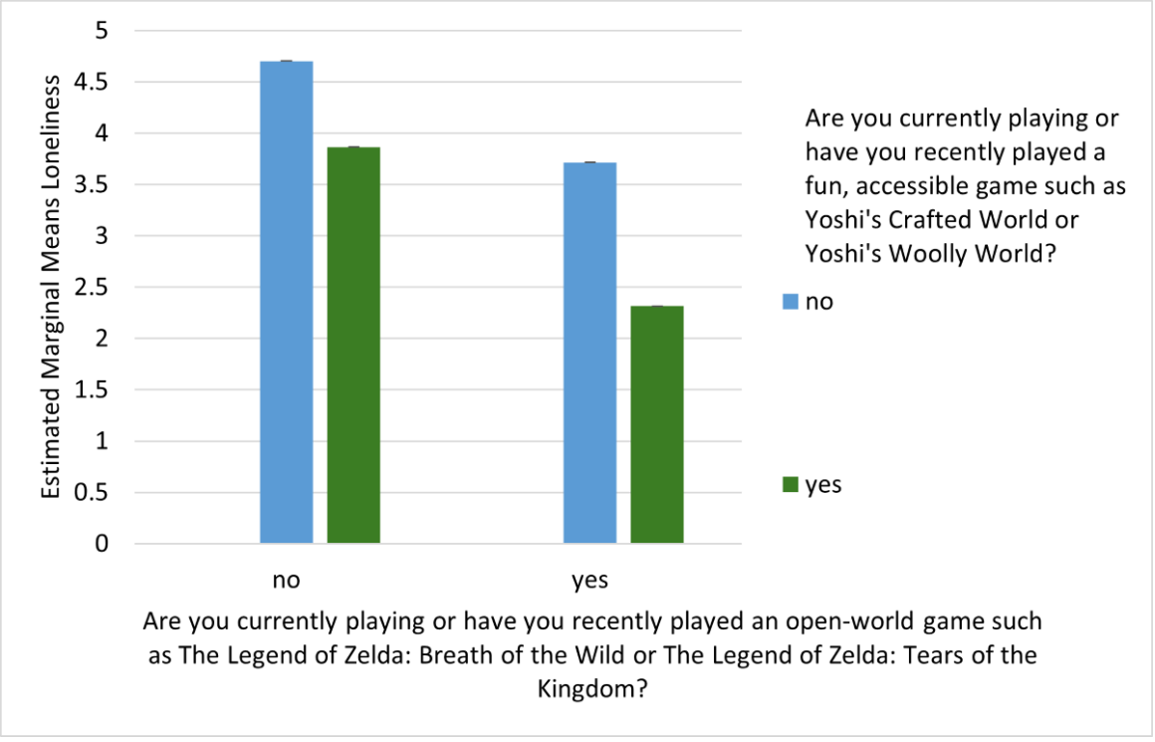

Supplement: Multimedia Appendix 2 [file games-v14-e89304-s002.png]
